# Immune-Genomic Evolution in AML Spontaneous Remission: A 66-Patient Pooled Analysis and Longitudinal Clonal Tracking

**DOI:** 10.3390/cancers18091398

**Published:** 2026-04-28

**Authors:** Yanping Sun, Mengyuan Chang, Jinlin Chen, Qirui Zhou, Fei Lu, Min Ji, Shaolei Zang, Jingjing Ye, Chunyan Ji

**Affiliations:** 1 Department of Hematology, Qilu Hospital, Cheeloo College of Medicine, Shandong University, Jinan 250012, China; syp215920@163.com (Y.S.); mengyuan_chang@mail.sdu.edu.cn (M.C.); 202435782@mail.sdu.edu.cn (J.C.); 202335706@mail.sdu.edu.cn (Q.Z.); lufei@qiluhospital.com (F.L.); jimin@sdu.edu.cn (M.J.); 15318806633@163.com (S.Z.); jichunyan@sdu.edu.cn (C.J.); 2 Shandong Key Laboratory of Hematological Diseases and Immune Microenvironment, Qilu Hospital, Cheeloo College of Medicine, Shandong University, Jinan 250012, China

**Keywords:** acute myeloid leukemia, spontaneous remission, clonal evolution, infection-associated immunity, tumor microenvironment, cytokine storm, interleukin-8

## Abstract

**Background/Objectives:** Spontaneous remission (SR) of acute myeloid leukemia (AML) offers unique clinical insights into host anti-tumor immunity. However, the comprehensive clinical landscape and molecular dynamics of blast clearance and subsequent relapse remain unclear. This study aimed to elucidate these dynamics. **Methods:** We conducted a two-phase observational study: a systematic pooled analysis of 66 adult AML SR cases (1990–2024) to define clinical triggers and outcomes and longitudinal molecular tracking of two institutional cases to map clonal shifts (with immune profiling for Patient 1 and genomic tracking for both). **Results:** In the pooled analysis, infection was the predominant trigger, accounting for 78.6% (95% CI: 65.6–88.4%) of SR events. The dataset showed male predominance and monocytic leukemia enrichment (57.6% [95% CI: 44.1–70.4%]), suggesting lineage-specific susceptibility. SR duration and relapse risk were independent of the infection trigger, AML subtype, or age. When integrated with these clinical patterns, institutional tracking was consistent with a biphasic evolutionary model: an acute IL-8 surge alongside NKT and CD4+ T cell activation coincided with blast clearance, as observed primarily in Patient 1. Subsequently, the emergence of *TP53* or *NRAS* mutations within persistent *DNMT3A*-mutated clones during relapse raised the hypothesis that unresolved chronic inflammation could potentially exert selective pressure favoring resistant subclones. Such interpretations remain correlational and require prospective validation. **Conclusions:** Our findings outline a clinical–evolutionary framework for AML SR. Remission durability likely relies on balancing acute immune activation with underlying clonal stability. These observational insights highlight complex immune-genomic crosstalk, generating hypotheses for future prospective investigations.

## Simple Summary

Acute myeloid leukemia is a severe blood cancer. Rarely, patients experience a “spontaneous remission,” where the leukemia disappears without chemotherapy, often after a severe infection. This phenomenon suggests that the body’s immune system can temporarily control the disease, but how this works—and why the cancer usually returns later—remains unclear. To explore this, we studied 66 historical cases and tracked the immune and genetic changes in two patients at our hospital. By combining our clinical observations with the existing scientific literature, we propose a theoretical “two-phase” hypothesis. We suggest that an initial severe infection might trigger an intense immune response that coincides with the clearing of leukemia cells. However, if the inflammation persists, this chronic stress might inadvertently create an environment that favors the survival of stronger, mutated leukemia cells, potentially leading to a relapse. Sharing these clinical observations benefits society by offering new clues for scientific inquiry. These insights provide researchers with specific directions to study how inflammation and genetic mutations interact over time in leukemia.

## 1. Introduction

Spontaneous remission (SR) of acute myeloid leukemia (AML) without cytotoxic therapy, although rare, has been reported for over a century [1], indicating that the host immune system can control disease in some situations. Early case reports mentioned that the leukemic burden sometimes decreases after febrile illnesses or transfusions, suggesting that pro-inflammatory cues trigger anti-leukemic immunity [2,3,4]. Although SR of AML occurs in less than 1% of patients, decades of observations suggest that infection-associated cytokine storms may contribute to disease regression [5]. Such systemic responses usually arise when pathogen-associated molecular patterns (PAMPs) interact with toll-like receptors (TLRs), connecting innate sensing to downstream effector activation. Inflammatory signals generated through this pathway may further mobilize and activate both innate and adaptive effector cells to clear AML [6,7].

However, inflammation is not always beneficial. Chronic signaling through interleukin (IL)-1β and IL-6 has been linked to leukemogenesis, which can hinder differentiation, while high IL-6 levels are associated with chemoresistance [8]. Similarly, IL-8 produced by leukemic blasts, especially those of monocytic lineage, supports survival and chemoresistance [9]. Furthermore, recent studies on immune-clonal interactions highlight how the inflammatory microenvironment can eventually foster an immunosuppressive niche, facilitating immune evasion and sub-clonal outgrowth [10,11,12].

Nevertheless, the underlying clinical and evolutionary dynamics by which acute inflammation induces SR while predisposing patients to relapse are poorly understood. To address this knowledge gap, we explicitly hypothesize that infection-triggered immunity acts as a temporary clearance force that, if not resolved, exerts selective pressure favoring clonal evolution. Therefore, we integrated a systematic pooled analysis of 66 historical cases with two institutional patients selected for their comprehensive longitudinal data, bridging broader clinical trajectories with complementary biological insights into immune and clonal dynamics.

## 2. Materials and Methods

### 2.1. Patient Selection and Ethics Statement

Two institutional cases of AML from Qilu Hospital of Shandong University were included. Both patients experienced SR following severe infections. Selection was based on the availability of comprehensive longitudinal data, including serial targeted NGS and acute-phase immune profiling, obtained during routine clinical management. The study was approved by the Medical Ethics Committee of Qilu Hospital of Shandong University and conducted in accordance with the Declaration of Helsinki.

### 2.2. Immune Profiling

Peripheral blood samples were collected in EDTA-anticoagulant tubes. For detailed immune profiling, lymphocyte subsets were analyzed using standardized 10-color flow cytometry panels (Supplementary Table S1) according to the EuroFlow protocol. In brief, whole blood was incubated with fluorochrome-conjugated antibodies tailored to the specific panels. Following staining, erythrocytes were lysed using a commercial lysing solution, and the cells were washed with phosphate-buffered saline (PBS) prior to acquisition. Data acquisition was performed on a Navios flow cytometer (Beckman Coulter, Brea, CA, USA).

Cytokine levels (including IL-8, IL-6, and TNF-α) in serum samples were quantified using the AimPlex multiplex flow cytometry detection technology (AimPlex Biosciences, Pomona, CA, USA) according to the manufacturer’s instructions. The analysis was performed using a BD FACSCanto II flow cytometer (BD Biosciences, San Jose, CA, USA).

### 2.3. Genomic Analysis

Somatic mutations were tracked using a targeted 500-gene next-generation sequencing (NGS) panel (Illumina NovaSeq, Illumina, San Diego, CA, USA). Library preparation, sequencing, and the standardized bioinformatics pipeline (encompassing read alignment to the human reference genome, variant calling, and annotation) were conducted by the certified clinical molecular diagnostics center affiliated with Qilu Hospital of Shandong University. Quality control criteria required a minimum sequencing depth of >500×. A 1% variant allele frequency (VAF) threshold was applied to reliably capture low-burden resistant subclones during the minimal residual disease (MRD) and early relapse phases. Chromosomal karyotyping was performed at diagnosis and relapse using G-banding (20 metaphases per sample).

### 2.4. Systematic Pooled Analysis: Search Strategy and Selection Criteria

In general accordance with PRISMA guidelines, we systematically searched PubMed and Web of Science for studies published between January 1990 and December 2024 using the terms (“acute myeloid leukemia” OR “AML”) AND (“spontaneous remission” OR “spontaneous regression”). Additionally, we integrated 57 historical cases compiled by Fan et al. (2021) [13] and identified nine new cases through our targeted search. Studies were included if they met the following criteria: (1) AML confirmed by morphology (≥20% blasts); (2) complete or partial remission without leukemia treatment; and (3) clinical data on possible triggers. We excluded pediatric cases (<14 years), acute promyelocytic leukemia, and cases lacking verifiable remission status. Data extracted included demographics, FAB subtype, cytogenetics/mutations, triggers, and remission/relapse outcomes.

### 2.5. Statistical Analysis

Continuous variables are summarized as ranges or interquartile ranges (IQR). Categorical variables are presented as frequencies and proportions, accompanied by 95% confidence intervals (CIs) calculated via the Clopper-Pearson exact binomial method. Remission duration was estimated using the Kaplan–Meier method, and group differences were compared using the Log-rank test. Effect sizes for duration comparisons are reported as Hazard Ratios (HR) with 95% CIs calculated using the Log-rank method. Fisher’s exact test was used to compare relapse rates, with effect sizes reported as Odds Ratios (OR) and 95% CIs. The correlation between age and remission duration was assessed using the Spearman rank correlation coefficient. All statistical tests were two-sided, and a *p*-value < 0.05 was considered statistically significant. Cases with missing information (“Not available”) for a given variable were excluded from the corresponding analysis. Analyses were performed using SPSS version 26.0 (IBM Corp, Armonk, NY, USA), R software (version 4.3.1, R Foundation for Statistical Computing, Vienna, Austria) and GraphPad Prism version 10.6 (GraphPad Software, Boston, MA, USA). Due to the nature of pooled isolated case reports lacking uniform baseline denominators, formal sensitivity analyses or statistical adjustments for publication bias were not performed. Therefore, all summary statistics derived from the pooled cohort represent unadjusted estimates.

## 3. Results

### 3.1. Systematic Pooled Analysis: Clinical Characteristics and Outcomes (N = 66)

To establish a comprehensive clinical framework for AML spontaneous remission (SR), we systematically analyzed a pooled dataset of 66 patients who achieved SR between 1990 and 2024. Importantly, the summary statistics presented herein represent unadjusted estimates, as the retrospective nature of isolated case reports precludes formal sensitivity analyses for publication bias.

**Figure 1:**
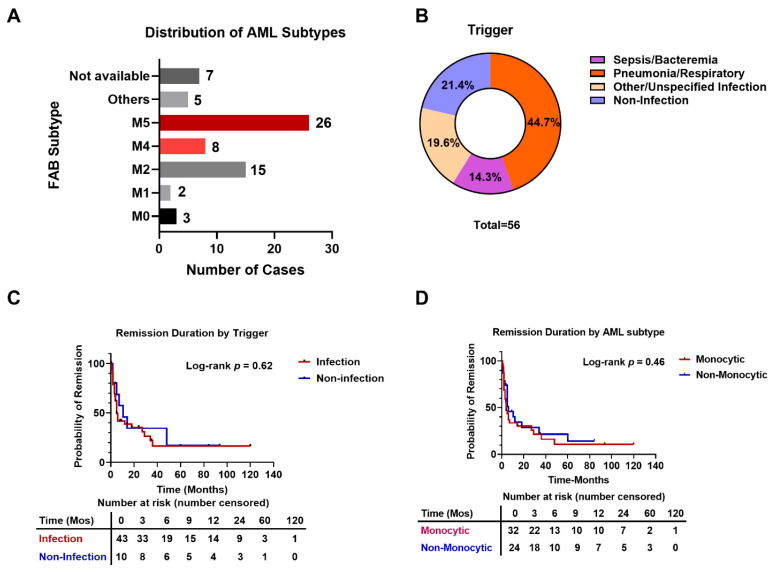
Clinical characteristics and remission outcomes of the 66-patient pooled cohort. (**A**) Distribution of FAB subtypes. Monocytic lineages (AML-M4 and AML-M5) are highlighted in red. (**B**) Frequencies of reported spontaneous remission precipitating events, categorized by infectious versus non-infectious etiologies. (**C**,**D**) Kaplan–Meier survival estimates comparing SR duration stratified by (**C**) infection trigger status and (**D**) AML subtype. *p*-values were calculated using the Log-rank test. Abbreviations: FAB, French-American-British classification; AML, acute myeloid leukemia.

The median age of the study population was 54 years (range: 17–83 years). The dataset showed a clear male predominance of 63.6% (42/66; 95% CI: 50.9–75.1%) (Table 1). In terms of FAB classification, we noted a striking enrichment of monocytic disease; AML-M5 (*n* = 26) and AML-M4 (*n* = 8) together accounted for 57.6% (34/59; 95% CI: 44.1–70.4%) of all cases with available AML subtype data (Figure 1A).

Infection was identified as the predominant trigger, accounting for 78.6% (44/56; 95% CI: 65.6–88.4%) of all SR events with available infectious status, with pneumonia (bacterial, fungal, or COVID-19) being the most frequently described etiology (Table 1, Figure 1B). Other reported triggers included blood transfusion, pregnancy termination, and withdrawal of immunosuppressive agents (Supplementary Table S6). SR was observed across the full range of cytogenetic risks, including in patients with complex karyotypes or adverse abnormalities, such as +8 and *TP53* alterations (Supplementary Table S6).

However, the median duration of SR was 5.0 months (IQR 2.25–18.0 months). Thus, although SR afforded a temporary response, it was seldom curative; most patients (75.0%, 48/64; 95% CI: 62.6–85.0%) ultimately relapsed, whereas only a minority (29.7%, 19/64; 95% CI: 18.9–42.4%) maintained remission beyond 12 months. Notably, while 97% (64/66) of the historical reports documented remission durations, longitudinal molecular tracking at relapse is rarely available (Supplementary Table S6).

### 3.2. Analysis of SR Duration and Relapse by Infection Trigger, AML Subtype, and Age

We next examined the relationship between specific variables and SR duration within the pooled dataset. Among 53 cases with available data, infection-triggered SR (*n* = 43) had a median duration of 5.0 months (IQR 3.0–29.0), whereas non–infection-triggered SR (*n* = 10) had a median duration of 10.5 months (IQR 5.0–48.0). This difference did not reach statistical significance (Log-rank *p* = 0.62; Hazard Ratio [HR] = 1.22, 95% CI: 0.56 to 2.66) (Figure 1C), likely due to the restricted sample size and unadjusted nature of the data. Relapse occurred in 69.8% of infection-triggered cases (30/43) and 70.0% of non–infection-triggered cases (7/10), with no significant difference (Fisher’s exact test, *p* = 1.00; Odds Ratio [OR] = 0.99, 95% CI: 0.18–6.94).

Furthermore, among 56 cases with available AML subtype data, there was no significant difference between monocytic (AML-M4/M5) and non-monocytic subtypes (Log-rank *p* = 0.46; HR = 1.25, 95% CI: 0.68–2.27) (Figure 1D). The median SR duration was 4.0 months (IQR 2.0–29.0) for monocytic cases and 6.0 months (IQR 3.0–34.0) for non-monocytic cases. Age likewise did not correlate with SR duration (Spearman r = 0.09, *p* = 0.49).

### 3.3. Longitudinal Tracking of Institutional Cases: Clinical Synopsis

To explore the clonal trajectories and available immune features underlying the infection-associated phenomena observed in the pooled dataset, we performed longitudinal tracking on two institutional cases. Both patients (aged 58 and 54 years) demonstrated spontaneous AML remission following severe infections, with subsequent relapse accompanied by clonal evolution. Importantly, while longitudinal genomic tracking was performed for both cases, comprehensive immune profiling was available only for Patient 1. Therefore, these observations reflect individual disease trajectories and should not be broadly generalized.

**Figure 2:**
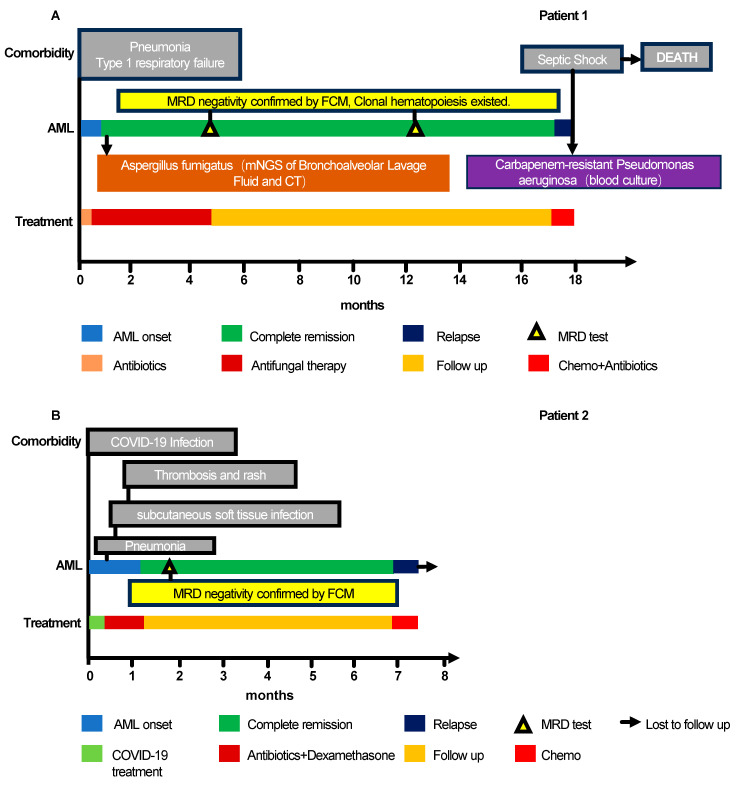
Longitudinal clinical and therapeutic timelines of the two institutional cases. (**A**) Patient 1 and (**B**) Patient 2. For each patient, the synchronized timeline aligns three main tracks: the onset and progression of infections or comorbidities (top row); the AML clinical course, incorporating key microbiological diagnostics and MRD monitoring (middle row); and the corresponding therapeutic interventions (bottom row). This layout visualizes the temporal relationship between severe infectious events, spontaneous morphological remission, and subsequent clinical relapse. Abbreviations: AML, acute myeloid leukemia; MRD, minimal residual disease.

Patient 1 was diagnosed with AML and identified with a *DNMT3A* p.R882P hotspot mutation (VAF 46.53%), *IDH2* p.H173 variants, and a *FLT3*-ITD mutation. The subsequent onset of invasive pulmonary aspergillosis coincided with rapid blast reduction (94% → 1%) without chemotherapy (Table 2, Supplementary Figure S1A–C). A repeat bone marrow examination on day 22 confirmed remission with a persistent *DNMT3A* p.R882P mutation at a reduced VAF of 6.28% (Supplementary Table S4). Patient 2 was diagnosed with de novo AML characterized by canonical *NPM1* and *DNMT3A* hotspot mutations. The patient experienced a spontaneous blast decline (35% → 4%) following the resolution of COVID-19, pneumonia, and a subcutaneous soft tissue infection (Table 2, Supplementary Figure S1D–F). The corresponding clinical trajectories are shown in Figure 2.

### 3.4. Infection-Associated Immune Dynamics

In Patient 1, immune profiling during the acute infection phase showed prominent dysregulation: the total lymphocyte proportion was diminished, whereas CD3+ T cells displayed a relative increase. Natural Killer T (NKT) cell ratios were elevated (11.16% vs. normal 3–8%). Activated CD4+ T cells and effector memory CD4+ T cells expanded, whereas regulatory T cells (Tregs) were reduced. We also noted a systemic loss of dendritic cells (mDCs/pDCs) and a skewed CD4+/CD8+ ratio (0.93) (Supplementary Table S2). Serum cytokine measurements showed more than a tenfold increase in IL-8 levels (199.18 pg/mL), along with raised IL-1β and IL-6 levels (Supplementary Table S3).

### 3.5. Mutational Trajectories and Longitudinal Clonal Dynamics

Longitudinal targeted sequencing revealed distinct mutational patterns during relapse. In Patient 1, AML recurrence was characterized by the emergence of a *TP53* p.Y234* truncating variant (VAF 20.57%) accompanied by complex karyotypic abnormalities (Table 2). Persistent *DNMT3A* p.R882P clones that survived the initial acute inflammatory phase likely acquired this *TP53* lesion (Supplementary Table S4). Patient 1 achieved a 16-month remission.

**Figure 3:**
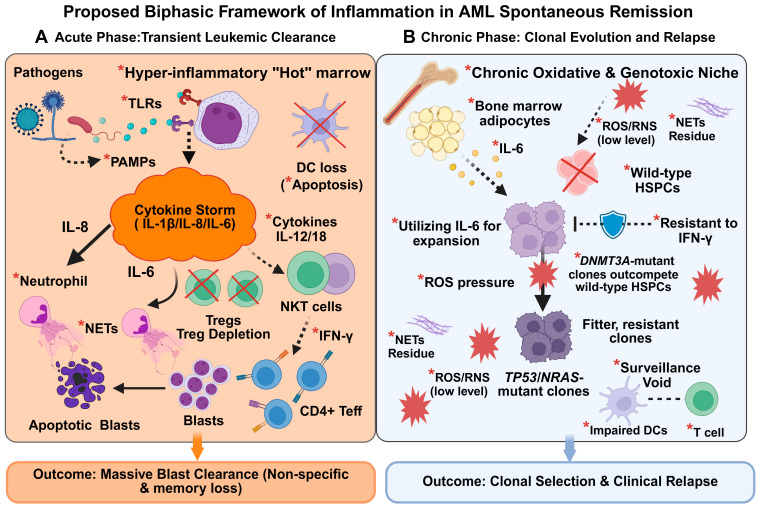
Proposed biphasic framework of inflammation in AML spontaneous remission and subsequent relapse. Solid lines denote variables observed in our cohort. Dashed lines and red asterisks (*) indicate hypothesized mechanisms based on the literature. (**A**) Solid lines show directly observed events, including the acute surge of inflammatory cytokines (IL-6, IL-8, IL-1β), regulatory T cell (Treg) depletion, dendritic cell (DC) loss, and the activation of NKT cells (green) and CD4+ effector T cells (Teff, blue) during the clearance of monocytic leukemic blasts (purple). Dashed lines illustrate hypothesized pathways where *PAMPs engage *TLRs to trigger the cytokine storm, and where IL-8 promotes neutrophils (pink) to release *NETs, exerting cytotoxicity against blasts. Additionally, it is inferred that high cytokines compensate for DC loss by activating NKT cells to secrete *IFN-γ, licensing CD4+ Teff for blast clearance. (**B**) Solid lines track the observed persistence of pre-leukemic *DNMT3A*-mutant clones (light purple) and the emergence of fitter subclones harboring *TP53* or *NRAS* mutations (dark purple). Dashed lines illustrate a hypothesized genotoxic niche characterized by persistent *ROS and *NETs residue. As wild-type HSPCs (pink/crossed out) are cleared, inferred mechanisms suggest surviving clones resist *IFN-γ-mediated depletion and utilize *IL-6 secreted by *bone marrow adipocytes (yellow) for expansion. This chronic stress is hypothesized to impose selective pressure, while the persistent impairment of DCs creates a surveillance void, failing to prime adaptive immunity against these evolving clones, ultimately facilitating clinical relapse. Abbreviations: AML, acute myeloid leukemia; DC, dendritic cell; HSPC, hematopoietic stem and progenitor cell; IFN-γ, interferon-γ; IL, interleukin; NET, neutrophil extracellular trap; NKT, natural killer T cell; PAMP, pathogen-associated molecular pattern; ROS, reactive oxygen species; Teff, effector T cell; TLR, Toll-like receptor; Treg, regulatory T cell.

In Patient 2 (6-month remission), relapse was defined by a newly acquired gain-of-function *NRAS* p.G12S mutation (VAF 16.17%) in a background of persistent *NPM1* p.W288fs and *DNMT3A* p.R882H mutations (Supplementary Table S5). During the relapse phase, fluctuating blast counts (61% → 27%) were observed over a ten-day interval without specific intervention. Taken together, these longitudinal observations document an evolutionary trajectory wherein persistent clones acquire secondary adaptations following severe inflammation (Figure 3).

## 4. Discussion

### 4.1. A Conceptual Biphasic Clinical–Evolutionary Framework: Infection-Immune-Clonal Interplay in AML Spontaneous Remission

Our systematic pooled analysis of 66 reported cases showed that infection (78.6%) was the most common trigger for SR, suggesting that pathogen-induced immunity can transiently overcome leukemic tolerance. Beyond the predominance of infection, the aggregated dataset revealed an enrichment of monocytic (M4/M5) AML and documented SR across the full spectrum of cytogenetic risk, including adverse karyotypes. Exploratory analyses further implied that the type of trigger (infectious vs. non-infectious), AML subtype, and age did not measurably influence SR duration or relapse risk. Rather than contradicting the immune-mediated nature of SR, this uniform lack of durable response indicates that while such variables may dictate the initiation of leukemia clearance, they are insufficient to establish lasting immunological memory. The clinical consequence of this transient control is exemplified by our institutional cases, which demonstrate the covert persistence and subsequent evolution of resistant subclones despite morphological remission. By integrating these epidemiological observations with the longitudinal tracking data from two patients (with immune profiling restricted to Patient 1), we tentatively propose a hypothesis-generating biphasic framework (Figure 3, dashed lines indicating inferred mechanisms). In this framework, infection triggers an immune response that initially drives leukemic clearance but subsequently exerts selective pressure favoring clonal evolution.

We postulate that monocytic leukemic blasts function as pathogen sensors during the acute phase of infection. Engagement of TLRs on these cells may precipitate a cytokine surge (IL-1β/IL-6/IL-8). This surge correlates with the infiltration of neutrophils and activates NKT cells together with CD4+ effector T-cell subsets, while diminishing Tregs. Consequently, a previously immunologically “cold” marrow may transition into a transiently “hot” environment that coincides with blast reduction. By contrast, in the subsequent chronic phase, persistent TLR signaling and unresolved inflammation may create an oxidative microenvironment. Building on the acute DC loss observed in Patient 1, we infer that prolonged DC dysfunction further aggravates this inflamed niche. As illustrated by the longitudinal genomic tracking in our cases, such a stress milieu may facilitate clonal evolution, with Darwinian selection favoring resistant subclones (e.g., *TP53*- or *NRAS*-mutant populations) within a genotoxic inflammatory niche. However, this literature-derived hypothesis requires direct functional validation.

### 4.2. Acute Infection Phase: Immune Activation and Leukemia Clearance

During the acute infection phase, the recognition of pathogens can engage TLRs and precipitate a systemic cytokine surge, characterized particularly by elevated IL-6 and IL-8 levels [14,15,16]. Our pooled dataset analysis implied that this inflammatory pattern reflects lineage-intrinsic and host-related influences. Given the enrichment of monocytic leukemia (AML-M5/M4, 57.6%) in the historical dataset, we posit that leukemic blasts may serve as primary pathogen sensors. Monocytic blasts constitutively display high TLR expression [17]. Once PAMPs ligate these receptors, these blasts may release large amounts of IL-6, IL-8, and IL-1β [8] in a “suicidal” inflammatory burst. This mechanism is consistent with data showing that TLR engagement drives NF-κB–dependent differentiation and p38 MAPK–mediated apoptosis in AML blasts [18,19]. Additionally, the male predominance (63.6%) in our pooled dataset is consistent with previous reports that men exhibit stronger pro-inflammatory responses to TLR stimulation than women [20]. This may lower the threshold for intense immune activation. However, given the restricted sample size of our pooled dataset, this sex-specific observation remains preliminary and requires validation in larger populations.

IL-8 appears to play a dual role in leukemia biology. Myeloid-derived IL-8 supports leukemic cell survival [21]. In contrast, IL-8 produced by endothelial cells can induce blast apoptosis in vitro [22], aligning with the rapid clearance seen in Patient 1. Meanwhile, IL-8 recruits neutrophils through CXCR1/2 and may prime them for phagocytosis and related effector functions [23,24]. Under hyper-inflammatory conditions, IL-8, together with pathogen- and damage-associated signals, may also enhance the formation of neutrophil extracellular traps (NETs) (NETosis) [25]. Although often linked to tissue injury, extracellular histones released during NETosis have been shown to exert potent and largely nonspecific cytotoxic effects [26]. Therefore, this mechanism reasonably contributes to the collateral clearance of stress-sensitive leukemic blasts during an acute cytokine storm.

In this context, the overall immune landscape exhibits a marked shift. The Treg depletion observed in Patient 1 is plausible because Tregs restrain anti-tumor immunity in AML, and their reduction can release inhibitory constraints on effector immune populations [27]. Notably, this increased activation arises despite reduced DC numbers. This observation aligns with evidence that severe infection induces DC apoptosis, impairing antigen presentation as sepsis progresses beyond the initial hyper-inflammatory phase [28,29]. We hypothesize that the cytokine storm may partly compensate for DC loss by directly activating NKT cells through IL-12 and IL-18 signaling, provoking interferon-γ (IFN-γ) release independently of TCR-mediated antigen recognition [30].

Accordingly, the expansion of NKT and effector memory CD4+ T cells (TEM) observed in Patient 1 points to a Th1-biased immune shift [31,32]. A recent study showed that IL-12-engineered NKT cells could maintain Th1 polarization over time [32], suggesting potential synergy with TEM populations [33]. Within our framework, activated NKT cells may provide an early source of IFN-γ. This supports an enlarging CD4+ TEM pool capable of exerting more sustained antileukemic activity [30,34]. However, this clearance mode has inherent limitations. The ongoing loss of DCs may push the immune system toward predominantly antigen-independent killing driven by excessive cytokines. Without functional DCs to prime new T cell responses, the host may fail to establish a robust, durable, and leukemia-specific immune memory [28]. Consequently, while a “hot” marrow environment can efficiently reduce bulk leukemic burden, the lack of immunological memory and residual inflammatory stress may create a surveillance gap that allows resistant clones to expand.

### 4.3. Chronic Inflammation Phase: Clonal Evolution and Relapse

Clinical observations in our two cases—one acquiring a *TP53* mutation and the other an *NRAS* mutation at relapse—demonstrate clear clonal evolution. To explain these genomic shifts within our framework, we postulate that unresolved inflammation exerts selective pressure favoring leukemic recurrence. As the initial cytokine storm settles into a chronic state, the bone marrow microenvironment theoretically undergoes pathological remodeling. Extrapolating from the acute phase, if IL-8 elevation persists, it may maintain neutrophil hyperactivation and NETosis while contributing to collateral tissue injury and fibrosis [35]. Within this proposed inflamed niche, mitochondrial dysfunction [36] and Nicotinamide Adenine Dinucleotide Phosphate (NADPH) oxidase activation [37] could generate excessive reactive oxygen/nitrogen species (ROS/RNS). This genotoxic milieu selectively induces DNA damage in proliferating cells [38].

The survival response to this putative inflammatory stress likely differs between normal and pre-leukemic populations. Although chronic inflammation may deplete normal hematopoietic stem cells, *DNMT3A*-mutant clones, present in both patients, appear capable of exploiting these signals. *DNMT3A* loss-of-function has been reported to render such clones resistant to IFN-γ-driven differentiation and depletion, allowing them to persist when wild-type cells are eliminated [39]. In addition, as presented by Zioni et al., these clones may preferentially expand in response to IL-6 produced by bone marrow adipocytes under inflammatory conditions [40]. However, since we did not directly evaluate the IL-6 pathway in our cases, its specific role requires further experimental confirmation. Such “inflammatory shielding” confers a competitive growth advantage, permitting the out-competition of normal hematopoietic stem and progenitor cells (HSPCs) and establishing a reservoir for further evolution [41].

As the inflammatory landscape evolves, this reservoir becomes vulnerable to additional oncogenic hits. Chronic inflammation has been shown to sustain *TP53*-mutant clones under DNA damage stress, enabling tolerance to genomic instability that would otherwise be lethal [42]. Prolonged fungal infection and cytokine exposure in Patient 1 may have favored the selection of *TP53* truncation. Meanwhile, the acquired *NRAS* p.G12S mutation in Patient 2 likely empowers resistant clones to hyperactivate survival signaling under ongoing inflammatory stress [43]. These clonal adaptations coincide with a postulated decline in broader immune function, notably prolonged dendritic cell impairment (deduced from the acute phase of Patient 1). In this sense, we consider the chronic phase as a critical intersection where inflammation may impose genotoxic stress and selective pressure on preleukemic clones while diminishing immune surveillance.

### 4.4. Implications for Future Mechanistic Research and Translational Exploration

Spontaneous remission (SR) serves as a “natural experiment.” It suggests that host immunity can temporarily clear bulk AML. However, this immune control is rarely sustained due to specific vulnerabilities, including dendritic cell (DC) loss, incomplete immune memory, and inflammatory niche remodeling. Our clinical–evolutionary framework therefore highlights a phase-informed approach for further scientific exploration. The theoretical goal is to recapitulate acute leukemic clearance while limiting chronic genotoxic pressure and constraining the pre-leukemic reservoir. As our biphasic hypothesis is conceptual and currently lacks direct in vivo functional validation, the phase-informed approaches discussed below represent avenues for future mechanistic research, rather than immediate clinical or therapeutic recommendations.

#### 4.4.1. Phase 1: Investigating the Balance Between Clearance and Inflammation

During the acute phase, a primary research objective is to understand how to decouple leukemic clearance from collateral tissue damage. Preclinical studies could investigate whether transiently modulating acute inflammatory pathways (such as the IL-1β/IL-8 axis) can preserve the antileukemic microenvironment while mitigating systemic toxicity. Additionally, given the observed DC impairment, exploring innate-like effector platforms in DC-deficient preclinical models may provide valuable insights into bypassing impaired antigen presentation [44,45].

#### 4.4.2. Phase 2: Exploring Mechanisms to Constrain the Pre-Leukemic Reservoir

The central translational question for this phase is determining how to disrupt the inflammatory niche that sustains adapted clones (e.g., *DNMT3A*-mutant reservoirs). Subsequent investigations might focus on how dampening specific signaling axes (e.g., the IL-6/JAK pathway) or reducing oxidative stress affects the competitive fitness of these clones. From a clinical research perspective, incorporating serial NGS surveillance into prospective observational cohorts could serve as a critical tool to longitudinally monitor these clonal trajectories, providing clinical correlates to the evolutionary dynamics proposed in our framework.

#### 4.4.3. Phase 3: Preclinical Evaluation of Immune Surveillance Restoration

Because DC loss impairs robust leukemia-specific immune memory, a key translational goal is to understand how to restore surveillance once acute inflammation subsides. Preclinical studies could test immunomodulatory strategies that directly enhance memory T-cell responses. For example, checkpoint modulation in post-inflammatory animal models may reveal how to convert a transient inflammatory clearance into durable disease control.

### 4.5. Limitations and Future Directions

Several limitations of this study must be acknowledged. First, the multi-omics observations stem from only two institutional cases, reducing generalizability. Comprehensive immune phenotyping was only available for Patient 1, restricting comparative analysis. Additionally, immunological measurements were derived from peripheral blood; systemic cytokine levels may not fully reflect local or time-restricted microenvironmental signaling within the bone marrow niche. Furthermore, molecular tracking relied on bulk targeted NGS. The absence of single-cell sequencing precludes the definitive resolution of complex clonal diversity and evolutionary trajectories.

Second, the statistics derived from the 66-case pooled analysis represent unadjusted estimates. These retrospective data are confounded by inherent study heterogeneity and publication bias, as dramatic remission cases are preferentially reported. Because isolated case reports lack uniform denominators, formal sensitivity analyses to adjust for this bias could not be performed. Consequently, these summary statistics likely overestimate remission duration and should be interpreted with caution. Moreover, the lack of standardized inclusion criteria and the restricted sample size reduce the statistical power for robust subgroup analyses.

Finally, the proposed biphasic framework integrates our clinical observations with mechanisms from the prior literature. Key intermediates within this model—including NET burden, oxidative stress, and DNA-damage signatures—were not experimentally validated in our cases. Without direct functional validation, alternative explanations for disease relapse—such as the natural, inflammation-independent expansion of pre-existing mutant clones—cannot be definitively excluded. Therefore, these results should be interpreted as hypothesis-generating rather than establishing causality.

To address these epidemiological limitations, future research must establish prospective cohorts with standardized inclusion criteria and uniform data acquisition. Within these cohorts, incorporating longitudinal paired marrow–blood sampling for immune profiling and single-cell sequencing is essential to resolve the temporal coupling between inflammatory programs, immune remodeling, and clonal trajectories. In parallel, in vitro functional assays and infection-mimicking experimental systems (e.g., inflammatory mouse models on a *DNMT3A*-mutant clonal hematopoiesis background) are needed to test these literature-derived mechanisms and validate the proposed clinical–evolutionary framework.

## 5. Conclusions

Infection-associated SR suggests the dual nature of host immunity, which appears to mediate profound leukemic clearance but subsequently foster recurrent disease. By integrating clinical, immunologic, and genomic observations, we propose a hypothesis-generating biphasic framework. Within this model, we postulate that acute immune activation (such as NKT-mediated responses) can temporarily compensate for DC loss, whereas subsequent chronic inflammation exerts selective pressure fostering resistant clones. Ultimately, this framework provides a foundation for future preclinical research. The theoretical goal is to preserve initial immune clearance while constraining relapse-promoting inflammation. Achieving this balance may help convert transient SRs into durable clinical control.

## Figures and Tables

**Table 1:** Comparison of Prior Evidence and Direct Longitudinal Evidence in Infection-Associated AML Spontaneous Remission.

Parameter	Prior Evidence (Indirect, Static)	Evidence in This Study (Direct, Longitudinal)	Implications and Advancement
Population Characteristics			
Demographics (Median age)	54 (17–83)	58/54	Confirms SR can occur across typical age range.
Sex	Male predominance (63.6%)	Both are Male	Suggests a sex-based predisposition, potentially linked to the known male bias in severe sepsis and cytokine storm intensity.
AML Subtype	Enriched for Monocytic lineage (M4/M5: 57.6%)	Both patients: AML-M5	Highlights the intrinsic susceptibility of monocytic leukemia to inflammation-associated remission.
Infection Association	78.6% (mostly pneumonia/bacteremia)	2/2 patients (COVID-19, fungal/bacterial)	Supports infection as a primary precipitating factor, with modern spectrum of pathogens.
Immune Profiling			-
Immune activation	Hypothesized (e.g., TNF-α/G-CSF)	Patient 1: IL-8 ↑ (199.1 pg/mL), IL-6 ↑, IL-1β ↑; Activated CD4+ ↑, NKT ↑, Treg ↓, DCs ↓	Provides longitudinal, quantitative evidence of a cytokine storm and specific immune cell shifts, moving beyond inference.
Clonal Evolution			
Clonal dynamics	Limited, static reports (e.g., *NPM1* loss)	Patient 1: *DNMT3A* p.R882P ↓, *IDH2* p.H173 ↓, *TP53* p.Y234 *; Patient 2: *DNMT3A* p.R882H ↑, *NPM1* p. W288fs ↑, *NRAS* p.G12S *	Longitudinal NGS tracking demonstrating active clonal selection concomitant with inflammation, revealing divergent evolutionary paths (*TP53* vs. *NRAS*)
Conceptual Framework	Immune suppression of clones	A biphasic framework: Acute immunity correlates with blast clearance, whereas chronic inflammation exerts selective pressure favoring evolution.	Proposes a dynamic clinical–evolutionary framework that contextualizes both remission and relapse, addressing the paradox of infection’s dual role.

Abbreviations: AML, acute myeloid leukemia; COVID-19, coronavirus disease 2019; DC, dendritic cell; *DNMT3A*, DNA methyltransferase 3 alpha; G-CSF, granulocyte colony-stimulating factor; IL, interleukin; NGS, next-generation sequencing; NKT, natural killer T cell; *NRAS*, neuroblastoma *RAS* viral oncogene homolog; SR, spontaneous remission; TNF-α, tumor necrosis factor-alpha; *TP53*, tumor protein p53; Treg, regulatory T cell. * Indicates a newly detected subclone that emerged at the time of clinical relapse; ↑, increase or up-regulation; ↓, decrease or down-regulation

**Table 2:** Comparative Clinical, Genomic, and Immunological Features of *TP53*-Mutant (Patient 1) vs. *NRAS*-Mutant (Patient 2) AML.

Category	Feature	Patient 1 (*TP53*-Mutant)	Patient 2 (*NRAS*-Mutant)
Clinical Timeline	Demographics	Age 58, Male	Age 54, Male
	Diagnosis	AML-M5	AML-M5
	Diagnosis Date	October 2022	May 2023
	Relapse Date	February 2024 (16 months post-remission)	December 2023 (6 months post-remission)
	Outcome	Deceased (sepsis)	Lost to follow-up
Disease Burden	Bone Marrow Blasts (%)	94 → 1 (remission) → 75 (relapse)	35 → 4 (remission) → 61 (relapse) → 27 (relapse)
	Immunophenotyping (%)	Aberrant blasts: 88.99 → <0.01→ 70.37	Aberrant blasts: 3.71 → <0.01→ 21.63
	Remission Status	CR, MRD < 0.01%	CR, MRD < 0.01%
Genetic Profile	Initial Mutations	*FLT3*-ITD (low VAF), *DNMT3A* p.R882P, *IDH2* p.H173	*NPM1* p.W288fs, *DNMT3A* p.R882H
	Relapse-Acquired Mutations	*TP53* p.Y234 *, complex karyotypic abnormalities (+8, del13q, add7p, add17p)	*NRAS* p.G12S *
	Predicted Functional Impact	Tumor suppressor loss (associated with genomic instability)	Oncogenic activation (associated with MAPK hyperactivation)
	VAF Dynamics (%)	*DNMT3A* p.R882P (46.53 → 40.8), *IDH2* p.H173 (43.67 → 36.67), *TP53* p.Y234 * (20.57)	*DNMT3A* p.R882H (35.77 → 48.18), *NPM1* p.W288fs (27.77 → 39.98), *NRAS* p. G12S * (16.17)
	Clonal Architecture	Evolution to a complex karyotype with multiple subclones	Stable clonal background
Immunology	Immune Subsets	Activated CD4+ T cells ↑, NKT ↑, Treg ↓, mDC/pDC undetectable	Not assessed
	Cytokines (pg/mL)	IL-1β 10.77, IL-6 50.6, IL-8 199.1	Not assessed
Infection Context	Infection Types	Invasive pulmonary aspergillosis	COVID-19, pneumonia, right buttock soft tissue infection
	Impact on Disease	Spontaneous remission → Relapse	Spontaneous remission → Relapse

Abbreviations: AML, acute myeloid leukemia; COVID-19, coronavirus disease 2019; CR, complete remission; MRD, minimal residual disease; *FLT3*-ITD, *FMS-like tyrosine kinase 3* internal tandem duplication; IL, interleukin; mDC, myeloid dendritic cell; MAPK, mitogen-activated protein kinase; NKT, natural killer T cell; pDC, plasmacytoid dendritic cell; Treg, regulatory T cell; VAF, variant allele frequency. * Indicates a newly detected subclone that emerged at the time of clinical relapse; ↑, increase or up-regulation; ↓, decrease or down-regulation

## Data Availability

The original contributions presented in the study are included in the article/Supplementary Materials. Further inquiries can be directed to the corresponding author.

## Supplementary Materials

The following supporting information can be downloaded at: https://www.mdpi.com/article/10.3390/cancers18091398/s1, Figure S1. Radiological progression of pneumonia in the institutional cases; Table S1. Flow cytometry panels for immune profiling of Patient 1; Table S2. Peripheral blood immune subsets in Patient 1; Table S3. Serum cytokine levels in Patient 1; Table S4. Clinical and genomic profile of Patient 1 during spontaneous remission and relapse; Table S5. Clinical and genomic profile of Patient 2 during spontaneous remission and relapse; Table S6. Summary of AML spontaneous remission cases published between 1990 and 2024 (*n* = 66). Furthermore, references [2,3,4,7,13] and [46,47,48,49,50,51,52,53,54,55,56,57,58,59,60,61,62,63,64,65,66,67,68,69,70,71,72,73,74,75,76,77,78,79,80,81,82,83,84,85,86,87,88,89,90,91,92,93,94,95,96] are also cited in the Supplementary Materials.
