# Supplementary material for: Immune-Genomic Evolution in AML Spontaneous Remission: A 66-Patient Pooled Analysis and Longitudinal Clonal Tracking
Source: Cancers (Basel). 2026 Apr 28;18(9):1398. doi: 10.3390/cancers18091398 (PMC13162684; doi:10.3390/cancers18091398)
Supplement: Supplementary file 1 [file cancers-18-01398-s001.zip › cancers-4230093-supplementary.pdf]

Supplementary Material

1 Supplementary Figures and Tables

1.1 Supplementary Tables

Supplementary Table S1. Flow cytometry panels for immune profiling of Patient 1

Panel	Antibody Combinations (Fluorochrome)	Target Cells
Panel 1	HLA-DR (FITC), CD25 (PE), CD4 (PerCP), CD45RO (APC), CD127 (PE-Cy7), CD16 (APC-H7), CD56 (BV421)	T-cell subsets, NK cells
Panel 2	CD183 (BV510), CD197 (BV605), CD45RA (BV650), HLA-DR (BV711), CD196 (BV786), CD38 (FITC), CD8 (PE)	T-cell activation markers
Panel 3	CD16 (APC), Lineage (FITC), CD1c (PE), CD11c (PerCP), CD370 (APC-H7), CD123 (BV421), HLA-DR (BV510)	Dendritic cell subsets
Panel 4	IgD (FITC), CD21 (PE), CD19 (PerCP), CD27 (APC), CD24 (PE-Cy7), CD38 (APC-H7), IgM (BV421)	B-cell maturation stages

Supplementary Table S2. Peripheral blood immune subsets in Patient 1

Category	Parameter	Result (%)	Reference Range	Status
**Total Lymphocytes**	Lymphocytes (% nucleated cells)	3.10	11.40–57.00	↓
**T Lymphocytes**	CD3+ T cells (% lymphocytes)	87.43	53.70–82.80	↑
	CD3+CD4+ T cells (% T cells)	35.28	46.20–78.00	↓
	CD3+CD8+ T cells (% T cells)	37.91	14.80–48.40	–
	CD3+CD4+/CD3+CD8+ (Th/Tc)	0.93	1.50-3.60	↓
	**NKT cells (% lymphocytes)**	11.16	3.00–8.00	↑

Category	Parameter	Result (%)	Reference Range	Status
	**Activated CD4+ T cells (% Th)**	47.10	3.60–31.40	↑
	**Effector CD4+ T cells (% Th)**	0.27	4.90-43.60	↓
	**Effector Memory CD4+ T cells (% Th)**	35.28	0.10–11.70	↑
	**Regulatory T cells (Tregs) (% Th)**	4.40	5.10–12.70	↓
**Dendritic Cells**	**Myeloid dendritic cells (% nucleated)**	0.00	0.10–1.70	↓
	**Plasmacytoid dendritic cells (% nucleated)**	0.00	0.00–0.40	–

Supplementary Table S3. Serum cytokine levels in Patient 1

Cytokine	Result (pg/mL)	Reference Range	Status
**IL-1β**	10.77	≤3.40	↑
**IL-2**	1.72	≤6.64	–
**IL-4**	0.92	≤4.19	–
**IL-5**	0.90	≤4.15	–
**IL-6**	50.66	≤11.09	↑
**IL-8**	199.18	≤15.71	↑
**IL-10**	1.43	≤4.50	–
**IL-12p70**	1.03	≤10.18	–

Cytokine	Result (pg/mL)	Reference Range	Status
**IL-17A**	0.02	≤4.74	–
**IL-17F**	0.72	≤4.66	–
**IL-22**	0.78	≤3.64	–
**TNF-α**	1.06	≤4.50	–
**TNF-β**	1.39	≤2.54	–
**IFN-γ**	0.76	≤4.43	–

Supplementary Table S4. Clinical and genomic profile of Patient 1 during spontaneous remission and relapse

Time Point	Marrow Blasts (%)	MRD by Flow Cytometry (%)	Genetic Mutations (VAF, %)	Karyotype	Clinical Phases/Remarks
2022-10-23 (Diagnosis)	94	88.99	*FLT3*-ITD (0.53), *DNMT3A* p.R882P (46.53), *IDH2* p.H173 (43.67)	46, XY [20]	AML diagnosed
2022-11-02 (Post infection)	12	4.84	*CEBPA* p.H195_insHP (5.66), *DNMT3A* p.R882P (4.47), *IDH2* p.H173 (4.76)	47, XY, -13, +mar1, +mar2[1]	During severe infection (Aspergillosis); onset of blast reduction

**Time Point**	**Marrow Blasts (%)**	**MRD by Flow Cytometry (%)**	**Genetic Mutations (VAF, %)**	**Karyotype**	**Clinical Phases/Remarks**
2022-11-14 (Remission)	1	<0.01	*DNMT3A* p.R882P (6.28)	46, XY [20]	CR achieved; pre-leukemic clone (*DNMT3A* p.R882P) persists
2023-03-14 (Remission)	Occasional	<0.01	*CEBPA* p.H195_insHP (2.14), *DNMT3A* p.R882P (3.35)	Not inspected	pre-leukemic clone (*DNMT3A* p.R882P) persists
2023-10-26 (Remission)	0	<0.01	*DNMT3A* p.R882P (4.25)	Not inspected	pre-leukemic clone (*DNMT3A* p.R882P) persists
2024-03-25 (Relapse)	75	70.37	*DNMT3A* p.R882P (40.8), *IDH2* p.H173 (36.67), *TP53* p.Y234* (20.57)	Main clone: 47, XY, +8, del(13q) [5] Subclone 1: 48, idem, +del(13q) [7] Subclone 2: 47, idem, add(17p) [2] Subclone 3: 47, idem, add(7p), add(17p) [3] Subclone 4: 46, XY [3]	Relapse with genomic instability (*TP53* mutation, complex karyotype)

*Indicates a newly detected subclone that emerged at the time of clinical relapse

Supplementary Table S5. Clinical and genomic profile of Patient 2 during spontaneous remission and relapse

Time Point	Marrow Blasts (%)	MRD by Flow Cytometry (%)	Genetic Mutations (VAF, %)	Karyotype	Clinical Phase/Remarks
2023-5-10 (Diagnosis)	35	Not inspected	Not inspected	Not inspected	Active disease, pre-infection
2023-5-15 (Post-infection)	11	3.71	*DNMT3A* p.R882H (35.77), *NPM1* p.W288fs (27.77)	46, XY [20]	Onset of spontaneous remission
2023-6-15 （Remission）	4	0.03	*DNMT3A* p.R882H (31.42), *NPM1* p.W288fs (26.07)	46, XY [20]	Complete remission
2023-7-6 (Remission)	No blasts	<0.01	Not inspected	Not inspected	Deep remission
2023-12-5 (Relapse)	61	Not inspected	Not inspected	Not inspected	Relapse diagnosed
2023-12-15 (Post relapse)	27	21.63	*DNMT3A* p.R882H (48.18), *NPM1* p.W288fs (39.98), *NRAS* p.G12S* (16.17)	46, XY [20]	Infection-associated partial blast clearance; Clonal evolution (*NRAS* p.G12S*)

*Indicates a newly detected subclone that emerged at the time of clinical relapse

Supplementary Table S6. Summary of AML spontaneous remission cases published between 1990 and 2024 (*n* = 66)

Reference	Age/Gender	AML Subtype	Key Genetic Alterations	Trigger/Intervention	Remission Duration	Outcome
(Okagawa et al., 1990)	47/Female	AML-M5	*+8*	No infection; Fever	12 months	Not available
(Fassas et al., 1991)	58/Male	AML-M4	*Not available*	Pneumocystis carinii pneumonia; Fever, Transfusion	6 months	Relapse
(Zhang et al., 1991)	20/Male	AML-M5	*Not available*	Malaria; Fever, Transfusion	3 months	Relapse
(Jimemez et al., 1993)	72/Female	AML-M0	*Triploid*	S.epidermidis pneumonia; Fever, Transfusion	5 months	Relapse
(Yan et al., 1993)	32/Male	AML-M2	*Not available*	Malaria, left lower limb infection; Fever	5 months	Relapse
(Musto et al., 1994)	49/Female	AML-M5	*Not available*	Laparotomy, mucosal ulcer; Fever	6 months	Relapse
(Delmer et al., 1994)	54/Female	AML-M4	*Normal karyotype*	Gram-negative bacilli sepsis; Transfusion	3 months	Relapse
(Delmer et al., 1994)	41/Female	AML-M5	*Normal karyotype*	No infection; Fever, Transfusion	14 months	Relapse
(Delmer et al., 1994)	48/Male	AML-M5	*t(8;21), -Y*	Gram-negative bacteria, Candida; Transfusion	36 months	Relapse
(Delmer et al., 1994)	56/Male	AML-M1	*Complex karyotype*	Tuberculosis (Lung and Liver); Transfusion	34 months	Relapse
(Hayatsu et al., 1994)	54/Male	AML-M2	*+8*	Pneumonia	Not available	Relapse
(Chen et al., 1995)	74/Female	AML-M5	*Complex karyotype*	Not available	7 months	Relapse
(Mitterbauer et al., 1996)	64/Male	AML-M5	*Not available*	Enterococcus pneumonia; Fever,	14 months	Continued Remission

Reference	Age/Gender	AML Subtype	Key Genetic Alterations	Trigger/Intervention	Remission Duration	Outcome
				Transfusion		
(Mitterbauer et al., 1996)	83/Male	AML-M2	*t(8;21)(q22;q22), del(7)(q22)*	Pneumonia; Fever, Transfusion	1 month	Relapse
(Zhao et al., 1998)	32/Male	AML-M2	*Not available*	Pharyngeal infection; Fever	84 months	Continued Remission
(Zhao et al., 1998)	21/Male	AML-M2	*t(8;21)*	Not available; Fever, Transfusion	60 months	Relapse
(Shimohakamada et al., 2001)	71/Female	AML-M2	*Complex karyotype*	Pneumonia; Fever, Transfusion	4 months	Complete remission
(Tzankov et al., 2001)	60/Female	AML-M1	*Normal karyotype*	Pulmonary Aspergillus; Fever, Transfusion	3 months	Relapse
(Chen et al., 2003)	34/Female	Myeloid Sarcoma	*Normal karyotype*	Not available; Fever, Transfusion	0.5 months	Relapse
(Fozza et al., 2004)	72/Male	AML-M0	*Complex karyotype*	Staphylococcus pneumonia, Candida; Fever, Transfusion	5 months	Relapse
(Maywald et al., 2004)	31/Male	AML-M5	*Normal karyotype*	Group G Streptococcus bacteremia; Fever	2 months	Relapse
(Sonneck et al., 2005)	67/Female	Not available	*Normal karyotype*	Not available ; Transfusion	20 months	Relapse
(Tsavaris et al., 2006)	64/Male	AML-M4	*Not available*	No infection; Fever	48 months	Relapse
(Al-Tawfiq and Al-Khatti, 2007)	47/Male	AML-M5	*Normal karyotype*	Clostridium septicum sepsis; Fever, Transfusion	4 months	Relapse
(Daccache et al., 2007)	83/Female	AML-M5	*+8*	Urinary tract infection; Fever, Transfusion	3 months	Relapse
(Trof et al., 2007)	28/Male	AML-M5	*Normal karyotype*	Sepsis: β -hemolytic Streptococcus; Fever, Transfusion	1 month	Relapse
(Trof et al.,	29/Male	AML-M2	*t(8;21), -Y*	Pneumonia; Fever,	6 months	Relapse

Reference	Age/Gender	AML Subtype	Key Genetic Alterations	Trigger/Intervention	Remission Duration	Outcome
2007)				Transfusion		
(Hudecek et al., 2008)	35/Female	Not available	*Complex karyotype including an 11q23/MLL*	Upper respiratory infection; Fever, Transfusion	8 months	Complete remission
(Nitin et al., 2008)	46/Male	AML-M5	*+8*	Liver abscess; Fever	2 months	Relapse
(Nitin et al., 2008)	72/Female	AML-M5	*Not available*	Not available	5 months	Relapse
(Nitin et al., 2008)	66/Female	AML-M4	*+8*	Pulmonary Candida; Fever, Transfusion	29 months	Relapse
(Marisavljevic et al., 2009)	63/Male	AML-M2	*del(6)(q21)*	No infection; Fever, Transfusion	5 months	Relapse
(Cai and Zhang, 2011)	31/Female	AML-M5	*Not available*	No infection; Fever, Transfusion	Not available	Not available
(Teng et al., 2011)	75/Male	Not available	*+8*	Pneumonia; Fever, Transfusion	5 months	Relapse
(Muller et al., 2004; Muller-Schmah et al., 2012)	61/Male	AML-M5	*t(9;11)(q21;q23)*	Pneumonia, Sepsis, Fever	120 months	Continued remission
(Liu et al., 2012)	21/Male	AML-M2	*Not available*	Lung infection; Fever	1 month	Relapse
(Xie et al., 2012)	42/Male	AML-M5	*Normal karyotype*	Pneumonia; Fever	27 months	Relapse
(Zeng et al., 2013)	34/Female	Myeloid Sarcoma	*Normal karyotype*	Not available; Fever, Transfusion	2 months	Relapse
(Zeng et al., 2013)	31/Male	AML-M2	*t(8;21), idem, del(9)*	Serratia pneumonia; Fever, Transfusion	2 months	Relapse
(Adam and Eltayeb, 2014)	35/Male	AML-M4	*Not available*	No infection; Fever, Transfusion	1.5 months	Relapse
(Kazmierczak et al., 2014)	77/Male	Not available	*48,XY,+13,+21/46,XY*	No infection; Fever, Transfusion	7 months	Relapse

Reference	Age/Gender	AML Subtype	Key Genetic Alterations	Trigger/Intervention	Remission Duration	Outcome
(Liu et al., 2015)	48/Male	AML-M2	*t(10;11)(q21;q23)*	Pulmonary Aspergillus, Liver infection; Fever	0.6 months	Complete Remission
(Camus et al., 2015)	74/Female	AML-M5	*Normal karyotype*	Pneumonia; Fever	2 months	Relapse
(Camus et al., 2015)	33/Male	AML-M5	*Normal karyotype*	Sepsis, Pulmonary Aspergillus; Fever	1.5 months	Relapse
(Camus et al., 2015)	24/Female	AML-M5	*Not available*	Not available; Fever	2 months	Relapse
(Bu, 2016)	30/Male	AML-M5	*t(9;11)(p22;q23)*	Lung infection; Fever	2 months	Relapse
(Mozafari et al., 2017)	53/Male	AML-M4	*Normal karyotype*	Klebsiella pneumoniae bacteremia; Fever, Transfusion	18 months	Continued Remission
(Hoshino et al., 2018)	49/Female	AML-M5	*t(8;16)(p11;p13)*	No infection; Fever	4 months	Complete Remission
(Kremer et al., 2018)	51/Male	AML-M5	*Complex karyotype*	No infection; Fever	2 months	Relapse
(Grunwald et al., 2019)	72/Male	Not available	*Normal karyotype*	Not available; Transfusion	12 months	Relapse
(Rautenberg et al., 2019)	59/Female	AML-M0	*47,XX,t(4;12),+mar*	Hand, foot and mouth disease	18 months	Relapse
(Suyama and Hasebe, 2019)	67/Male	AML-M5	*Complex karyotype*	Not available; Fever, Transfusion	0.7 months	Relapse
(Bradley et al., 2020)	58/Male	Not available	*Normal karyotype*	Acute colitis; Fever, Transfusion	24 months	Continued Remission
(Helbig et al., 2020)	40/Male	Not available	*Normal karyotype*	Hip Staphylococcus aureus infection; Fever, Transfusion	14 months	Continued Remission
(Waller et al., 2020)	66/Male	AML-M2	*Normal karyotype*	No infection	10.5 months	Relapse
(Li et al., 2021)	78/Male	AML-M4	*Not available*	Not available	4 months	Relapse

Reference	Age/Gender	AML Subtype	Key Genetic Alterations	Trigger/Intervention	Remission Duration	Outcome
(Fan et al., 2021)	48/Male	AML-M5	*t(9;11)(p22;q23)*	Pneumonia; Fever, Transfusion	35 months	Continued Remission
(Sun et al., 2022)	63/Male	AML-M4	*RUNX1-RUNX1T1, FLT3-TKD Karyotype: 48, XY, t(8;21), +13[9]/49, idem, +mar [9]*	GI infection, skin lesions Antibiotics, glucocorticoids	CR1:51 days, CR2:54 days	Relapse
(Koedijk et al., 2023)	17/Male	Preleukemic clone	*MLLT10::UBE4A, KMT2A::MLLT10 KRAS mutation, chr 17p deletion*	COVID-19 pneumonia Antibiotics, antifungals, transfusion	9 months	Complete remission
(Miladinovic and Klusmann, 2023)	70/Male	AML-M2	*IDH2, WT1 mutations Karyotype: 46,XY*	H1N1 infection Oseltamivir	60 months	Continued Remission
(Imataki et al., 2023)	80/Female	AML-M2	*del(5q)*	Pulmonary NTM/aspergillosis Triple anti-Mycobacterium, voriconazole	CR1:7 months, CR2:5 months	Relapse
(Barkhordar et al., 2022)	57/Female	AML-M2	*KMT2A rearrangement Negative NPM1, FLT3-ITD/TKD*	COVID-19 pneumonia Remdesivir, dexamethasone, transfusion	5 months	Complete remission
(Martinez-Diez et al., 2022)	71/Male	AML secondary to MPN	*JAK2V617F, TP53R248Q, U2AF1Y158_159dup del(5q), del(13q)*	Upper respiratory infection Antibiotics	5 months	Relapse
(Armstrong et al., 2023)	55/Male	Myeloid sarcoma	*KMT2A-MLLT3 Karyotype: trisomy 8, t(9;11)*	None	2 years	Continued remission
(Wu et al., 2024)	70/Female	AML-M5	*RUNX1F416Rfs, IDH1R132C, SRSF2P95L Normal karyotype*	Staphylococcus bacteremia Antibiotics, antifungals	4 months	Relapse
(Zhang et al., 2024)	30/Female	AML-M5	*NPM1 mutation (201%) Karyotype: 46,XX*	Pregnancy termination	7.8 years	Continued Remission

References

1. Okagawa K, Kosaka M, Mima N, et al. Spontaneous complete remission in a patient with acute monocytic leukemia. *Rinsho Ketsueki* (1990) 31(11):1872－1877.

2. Fassas A, Sakellari I, Anagnostopoulos A, et al. Spontaneous remission of acute myeloid leukemia in a patient with concurrent Pneumocystis carinii pneumonia. *Nouv Rev Fr Hematol* (1991) 33(5):363 – 364.

3. Zhang Z, Zhang M. Spontaneous remission of acute leukemia after plasmodium infection: a case report. *Zhonghua Nei Ke Za Zhi* (1991) 30(11):731.

4. Jimenez C, Ribera JM, Abad E, et al. Increased serum tumour necrosis factor during transient remission in acute leukaemia. *Lancet* (1993) 341(8860):1600. doi: 10.1016/0140-6736(93)90739-4

5. Yan S. Spontaneous complete remission of acute leukemia after left lower limb abscess: a case report. *J Clin Hematol (China)* (1993) (4):179.

6. Musto P, D'Arena G, Melillo L, et al. Spontaneous remission in acute myeloid leukaemia: a role for endogenous production of tumour necrosis factor and interleukin-2? *Br J Haematol* (1994) 87(4):879 – 880.

7. Delmer A, Heron E, Marie JP, et al. Spontaneous remission in acute myeloid leukaemia. *Br J Haematol* (1994) 87(4):880 – 882. doi: 10.1111/j.1365-2141.1994.tb06762.x

8. Hayatsu K, Nagai K, Abe A, et al. Complete remission during administration of rhG-CSF in acute myeloblastic leukemia with pneumonia. *Rinsho Ketsueki* (1994) 35(1):59 – 64.

9. Chen Z. Spontaneous remission of acute myeloid leukemia. *Foreign Med Blood Transfus Hematol* (1995) (1):49 – 50.

10. Mitterbauer M, Fritzer-Szekeres M, Mitterbauer G, et al. Spontaneous remission of acute myeloid leukemia after infection and blood transfusion associated with hypergammaglobulinaemia. *Ann Hematol* (1996) 73(4):189 – 193. doi: 10.1007/s002770050226

11. Zhao L, Hu S, Li Q, et al. Spontaneous remission of acute myeloid leukemia after infection: 2 cases report. *J Intern Intensive Med* (1998) 4(2):95 – 96.

12. Shimohakamada Y, Shinohara K, Fukuda N. Remission of acute myeloblastic leukemia after severe pneumonia treated with high-dose methylprednisolone. *Int J Hematol* (2001) 74(2):173 – 177. doi: 10.1007/BF02982001

13. Tzankov A, Ludescher C, Duba HC, et al. Spontaneous remission in a secondary acute myelogenous leukaemia following invasive pulmonary aspergillosis. *Ann Hematol* (2001) 80(7):423 – 425. doi: 10.1007/s002770100300

14. Chen T, Li P, Chen Q, et al. Granulocytic sarcoma developing into acute leukemia with spontaneous remission followed by extramedullary relapse: a case report and literature review. *J Clin Hematol* (2003) 16(4):162 – 164.

15. Fozza C, Bellizzi S, Bonfigli S, et al. Cytogenetic and hematological spontaneous remission in a case of acute myelogenous leukemia. *Eur J Haematol* (2004) 73(3):219 – 222. doi: 10.1111/j.1600-0609.2004.00281.x

16. Maywald O, Buchheidt D, Bergmann J, et al. Spontaneous remission in adult acute myeloid leukemia in association with systemic bacterial infection—case report and review of the literature. *Ann Hematol* (2004) 83(3):189 – 194. doi: 10.1007/s00277-003-0741-y

17. Sonneck K, Mannhalter C, Krauth MT, et al. An unusual case of myelodysplastic syndrome with prolonged clonal stability, indolent clinical course over a decade, and spontaneous regression of AML in the terminal phase. *Eur J Haematol* (2005) 75(1):73 – 77. doi: 10.1111/j.1600-0609.2005.00423.x

18. Tsavaris N, Kopterides P, Kosmas C, et al. Spontaneous remission of acute myeloid leukemia associated with GnRH agonist treatment. *Leuk Lymphoma* (2006) 47(3):557 – 560. doi: 10.1080/10428190500343126

19. Al-Tawfiq JA, Al-Khatti AA. Spontaneous remission of acute monocytic leukemia after infection with Clostridium septicum. *Int J Lab Hematol* (2007) 29(5):386 – 389. doi: 10.1111/j.1365-2257.2006.00846.x

20. Daccache A, Kizhakekuttu T, Siebert J, et al. Hematologic and cytogenetic spontaneous remission in acute monocytic leukemia (FAB M5b) with trisomy 8. *J Clin Oncol* (2007) 25(3):344 – 346. doi: 10.1200/JCO.2006.08.8500

21. Trof RJ, Beishuizen A, Wondergem MJ, et al. Spontaneous remission of acute myeloid leukaemia after recovery from sepsis. *Neth J Med* (2007) 65(7):259 – 262.

22. Hudecek M, Bartsch K, Jäkel N, et al. Spontaneous remission of acute myeloid leukemia relapse after hematopoietic cell transplantation in a high-risk patient with 11q23/MLL abnormality. *Acta Haematol* (2008) 119(2):111 – 114. doi: 10.1159/000121827

23. Nitin J, Julie H. Spontaneous Remission of Acute Myeloid Leukemia: Report of Three Cases and Review of the Literature. *Clin Leuk* (2008) 2(1):64 – 67.

24. Marisavljevic D, Markovic O, Zivkovic R. An unusual case of smoldering AML with prolonged indolent clinical course and spontaneous remission in the terminal phase. *Med Oncol* (2009) 26(4):476 – 479. doi: 10.1007/s12032-008-9153-0

25. Cai Q, Zhang W. Spontaneous remission of pregnancy complicated with acute monocytic leukemia: a case report. *Hebei Med* (2011) 33(1):90.

26. Teng CJ, Yang CF, Gau JP, et al. Spontaneous remission in acute myelogenous leukemia evidenced by cytogenetic changes. *Ann Hematol* (2011) 90(8):981 – 983. doi: 10.1007/s00277-010-1120-0

27. Müller CI, Trepel M, Kunzmann R, et al. Hematologic and molecular spontaneous remission following sepsis in acute monoblastic leukemia with translocation (9;11): a case report and review of the literature. *Eur J Haematol* (2004) 73(1):62–66. doi: 10.1111/j.1600-0609.2004.00248.x

28. Muller-Schmah, C., Solari, L., Weis, R., Pfeifer, D., Scheibenbogen, C., Trepel, M., et al. (2012). Immune response as a possible mechanism of long-lasting disease control in spontaneous remission of MLL/AF9-positive acute myeloid leukemia. *Ann Hematol* 91(1), 27-32. doi: 10.1007/s00277-011-1332-y.

29. Liu C, Chen Z, Wang H, et al. Spontaneous remission of acute myeloid leukemia: a case report. *J Clin Hematol* (2012) 25(3):328.

30. Xie W, Zhao Y, Cao L, et al. Cutaneous blastic plasmacytoid dendritic cell neoplasm occurring after spontaneous remission of acute myeloid leukemia: a case report and review of literature. *Med Oncol* (2012) 29(4):2417–2422. doi: 10.1007/s12032-012-0215-y

31. Zeng Q, Yuan Y, Li P, et al. Spontaneous remission in patients with acute myeloid leukemia with t（8;21）or cutaneous myeloid sarcoma: two case reports and a review of the literature ［J］.Intern Med, 2013, 52 （11）:1227- 1233. DOI: 10.2169/ internalmedicine.52.9505.

32. Adam M, Eltayeb A. Spontaneous remission in acute myeloid leukemia: A Case Report. *Gulf J Oncolog* (2014) 1(15):84–86.

33. Kazmierczak M, Szczepaniak A, Czyż A, et al. Spontaneous hematological remission of acute myeloid leukemia. *Contemp Oncol (Pozn)* (2014) 18(1):67–69. doi: 10.5114/wo.2013.38915

34. Liu X, Yang L, Wen S, et al. Spontaneous complete remission of acute myeloid leukemia with t(10;11)(q22;q23) rearrangement: a case report and review of literature. *Chin J Hematol* (2015) 36(8):662–665.

35. Camus V, Etancelin P, Jardin F, et al. Spontaneous remission in three cases of AML M5 with NPM1 mutation. *Clin Case Rep* (2015) 3(11):955–959. doi: 10.1002/ccr3.408 [34]

36. Bu F. Spontaneous remission of acute myeloid leukemia: a case report and review of literature. Wuhan: Huazhong University of Science and Technology (2016).

37. Mozafari R, Moeinian M, Asadollahi Amin A. Spontaneous Complete Remission in a Patient with Acute Myeloid Leukemia and Severe Sepsis. *Case Rep Hematol* (2017) 2017:9593750. doi: 10.1155/2017/9593750

38. Hoshino T, Taki T, Takada S, et al. Spontaneous remission of adult acute myeloid leukemia with t(8;16)(p11;p13)/MOZ-CBP fusion. *Leuk Lymphoma* (2018) 59(1):253–255. doi: 10.1080/10428194.2017.1320712

39. Kremer B, Tsai DE, Debonera F, et al. Spontaneous remission of acute myeloid leukemia after discontinuation of lenalidomide. *Leuk Lymphoma* (2018) 59(3):743–745. doi: 10.1080/10428194.2017.1347652

40. Grunwald, V.V., Hentrich, M., Schiel, X., Dufour, A., Schneider, S., Neusser, M., et al. (2019). Patients with spontaneous remission of high-risk MDS and AML show persistent preleukemic clonal hematopoiesis. *Blood Adv* 3(18)**,** 2696-2699. doi: 10.1182/bloodadvances.2019000265.

41. Rautenberg C, Kaivers J, Germing U, et al. Spontaneous remission in a patient with very late relapse of acute myeloid leukemia 17 years after allogeneic blood stem cell transplantation. *Eur J Haematol* (2019) 103(2):131 – 133. doi: 10.1111/ejh.13245

42. Suyama T, Hasebe K. Spontaneous remission of acute monocytic leukemia with trisomy 8 and trisomy 18. *J Clin Exp Hematop* (2019) 59(2):96 – 97. doi: 10.3960/jslrt.19005

43. Bradley T, Zuquello RA, Aguirre LE, et al. Spontaneous remission of acute myeloid leukemia with NF1 alteration. *Leuk Res Rep* (2020) 13:100204. doi: 10.1016/j.lrr.2020.100204

44. Helbig D, Quesada AE, Xiao W, et al. Spontaneous Remission in a Patient With Acute Myeloid Leukemia Leading to Undetectable Minimal Residual Disease. *J Hematol* (2020) 9(1 – 2):18 – 22. doi: 10.14740/jh606

45. Waller DD, Monczak Y, Michel RP, et al. Spontaneous remission and clonal evolution in lenalidomide associated secondary AML. *Leuk Lymphoma* (2020) 61(7):1724 – 1727. doi: 10.1080/10428194.2020.1725504

46. Li X, Gan X, Fu H, et al. Spontaneous remission of acute myeloid leukemia secondary to multiple myeloma: a case report and literature review. *Leuk Lymphoma* (2021) 30(1):50 – 52. doi: 10.3760/cma.j.cn115356-20200421-00102

47. Fan WJ, Xu TT, Sang LN, et al. A case of spontaneous remission of acute myeloid leukemia with MLL-AF9 rearrangement and abnormal liver function. *Zhonghua Xue Ye Xue Za Zhi* (2021) 42(10):851 – 857. doi: 10.3760/cma.j.issn.0253-2727.2021.10.010

48. Sun, X.Y., Yang, X.D., Yang, X.Q., Ju, B., Xiu, N.N., Xu, J., et al. (2022). Antibiotic and glucocorticoid-induced recapitulated hematological remission in acute myeloid leukemia: A case report and review of literature. *World J Clin Cases* 10(22)**,** 7890-7898. doi: 10.12998/wjcc.v10.i22.7890.

49. Koedijk JB, van Beek TB, Vermeulen MA, et al. Case Report: Immune dysregulation associated with long-lasting regression of a (pre)leukemic clone. *Front Immunol* (2023) 14:1280885. doi: 10.3389/fimmu.2023.1280885

50. Miladinovic M, Klusmann JH. Influenza A (H1N1) virus induced long-term remission in a refractory acute myeloid leukaemia. *Br J Haematol* (2023) 202(4):713 – 714. doi: 10.1111/bjh.18911

51. Imataki O, Ishida T, Kida JI, et al. Repeated spontaneous remission of acute myeloid leukemia in response to various infections: a case report. *BMC Infect Dis* (2023) 23(1):215. doi: 10.1186/s12879-023-08108-z

52. Barkhordar M, Rostami FT, Yaghmaie M, et al. Spontaneous Complete Remission of Acute Myeloid Leukemia in the Absence of Disease-Modifying Therapy following Severe Pulmonary Involvement by Coronavirus Infectious Disease-19. *Case Rep Hematol* (2022) 2022:2603607. doi: 10.1155/2022/2603607

53. Martinez-Diez Y, Franganillo-Suarez A, Salgado-Sanchez R, et al. Spontaneous Remission of Acute Myeloid Leukemia: A Case Report. *Medicina (Kaunas)* (2022) 58(7):921. doi: 10.3390/medicina58070921

54. Armstrong C, Au WY, Ma ESK, et al. Sustained spontaneous remission in KMT2A-MLLT3 mutated myeloid sarcoma. *Ann Hematol* (2023) 102(5):1271 – 1274. doi: 10.1007/s00277-023-05173-x [54]

55. Wu X, Zhao J, Chen Y, et al. Transient Spontaneous Remission of Acute Myeloid Leukemia with Mutated RUNX1: A Rare Report. *Indian J Hematol Blood Transfus* (2024) 40(3):545 – 546. doi: 10.1007/s12288-024-01758-2 [55]

56. Zhang R, Zhang TS, Pan YL, et al. Spontaneous remission of acute myeloid leukemia with NPM1 mutation during pregnancy: a case report. *Zhonghua Xue Ye Xue Za Zhi* (2024) 45(9):876 – 877. doi: 10.3760/cma.j.cn121090-20230905-00103

1.2 Supplementary Figures

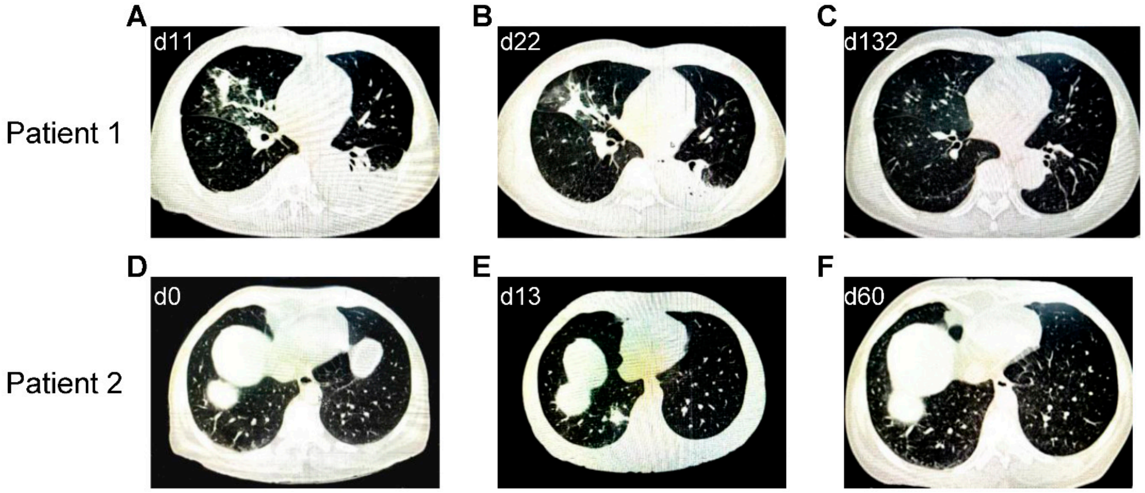

Supplementary Figure S1. Radiological progression of pneumonia in the institutional cases.

Thoracic CT scans illustrate the temporal progression of pneumonia in Patient 1 (Panels A-C) and Patient 2 (Panels D-F), with imaging time points annotated as days post-AML diagnosis (d = days).
